# Physiological Mechanisms Regulating Lens Transport

**DOI:** 10.3389/fphys.2021.818649

**Published:** 2021-12-23

**Authors:** Adrienne A. Giannone, Leping Li, Caterina Sellitto, Thomas W. White

**Affiliations:** ^1^Master of Science Program, Department of Biochemistry and Cell Biology, Stony Brook University, Stony Brook, NY, United States; ^2^Department of Physiology and Biophysics, School of Medicine, Stony Brook University, Stony Brook, NY, United States

**Keywords:** connexin, aquaporin, TRPV1, TRPV4, NKCC, Na^+^/K^+^-ATPase, lens

## Abstract

The transparency and refractive properties of the lens are maintained by the cellular physiology provided by an internal microcirculation system that utilizes spatial differences in ion channels, transporters and gap junctions to establish standing electrochemical and hydrostatic pressure gradients that drive the transport of ions, water and nutrients through this avascular tissue. Aging has negative effects on lens transport, degrading ion and water homeostasis, and producing changes in lens water content. This alters the properties of the lens, causing changes in optical quality and accommodative amplitude that initially result in presbyopia in middle age and ultimately manifest as cataract in the elderly. Recent advances have highlighted that the lens hydrostatic pressure gradient responds to tension transmitted to the lens through the Zonules of Zinn through a mechanism utilizing mechanosensitive channels, multiple sodium transporters respond to changes in hydrostatic pressure to restore equilibrium, and that connexin hemichannels and diverse intracellular signaling cascades play a critical role in these responses. The mechanistic insight gained from these studies has advanced our understanding of lens transport and how it responds and adapts to different inputs both from within the lens, and from surrounding ocular structures.

## Introduction

**FIGURE 1:**
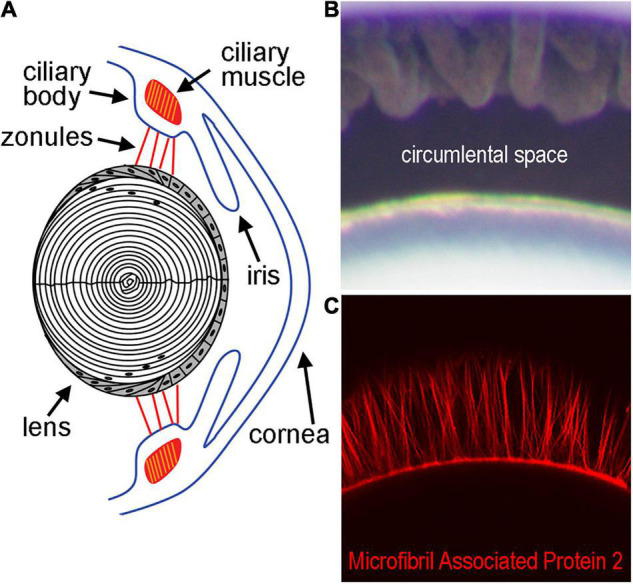
Diagram of the lens and supporting structures. **(A)** The anterior surface of the lens rests directly behind the iris and is attached by the Zonules of Zinn to the ciliary body. The Zonules of Zinn transduce tension generated by the ciliary muscle to the lens equator during the process of accommodation. **(B)** The area between the lens and ciliary body containing the zonules in a mouse eye, also known as the circumlental space, as visualized by light microscopy. **(C)** Fluorescent labeling of the zonules using an antibody against Microfibril Associated Protein-2 visualized by confocal microscopy.

The ocular lens is an avascular, non-innervated, and transparent structure that functions primarily in light transmission and refraction (Donaldson et al., 2017). Within the eye, the lens is suspended by the ciliary body via the zonules of Zinn (Figure 1), a fibrillar network (Raviola, 1971; Shi et al., 2013). The zonules transduce force from the ciliary muscle onto the lens during a process known as accommodation, which allows the eye to shift visual focus from far to nearby distances (Ott, 2006; Khan et al., 2018). Recently, forces transmitted through the zonules have been linked to changes in the hydrostatic pressure gradient that directs lens nutrient delivery and waste removal, even in the absence of accommodation (Gao et al., 2011; Chen et al., 2019). This pressure gradient acts in lieu of blood flow and helps sustain the ocular properties of the lens involved in transparency and light refraction (Mathias et al., 2007). The cells of the lens control the magnitude of the pressure gradient through modulation of the activity of pumps, channels, and other transport proteins to ensure that homeostasis in each region of the lens is maintained (Gao et al., 2011; Delamere et al., 2020).

Structurally, the lens is an elastic biconvex ellipsoid filled with concentric layers of cells that have different topologies on the anterior and posterior surfaces (McAvoy et al., 1999). At the equator, the epithelial cells lining the anterior surface differentiate into fiber cells, which elongate to span from beneath the anterior epithelium all the way to the posterior surface. As fiber cells mature, they lose their mitochondria, nuclei, ribosomes, and other organelles (McAvoy, 1978). Additionally, differentiation changes the content of fiber cell proteins, significantly increasing a class of lens-specific soluble proteins, called crystallins (Piatigorsky, 1981). Crystallin expression increases the refractive index of the lens, with the highest concentration of crystallin proteins in the central differentiated cells (Bassnett et al., 2011). The nucleus contains highly differentiated mature fiber cells (MFs) while the cortex contains differentiating fiber cells (DFs). These cells form concentric and organized layers and have a tight packing due to their elongated hexagonal shape (Bassnett and Costello, 2017). As the lens grows, the ends of newer fibers contact adjacent fibers at the poles, creating sutures down the central axis of the lens (Kuszak et al., 2004). The transparent and refractive properties of the lens are dependent on fiber cell specialization and maintenance. Since fiber cells do not have organelles and lack blood flow, they rely on transport proteins and hydrostatic pressure gradients for nutrient delivery and waste removal to maintain their transparent ocular properties.

Recent advances have broadened our understanding of how the microcirculatory system functions and fails. In this review, we discuss how this intricate network can change as a result of age and oxidative stress. We will also highlight how control of this network is assimilated into the rest of the eye through inputs from the ciliary muscle and zonules of Zinn.

## The Lens Circulation

**FIGURE 2:**
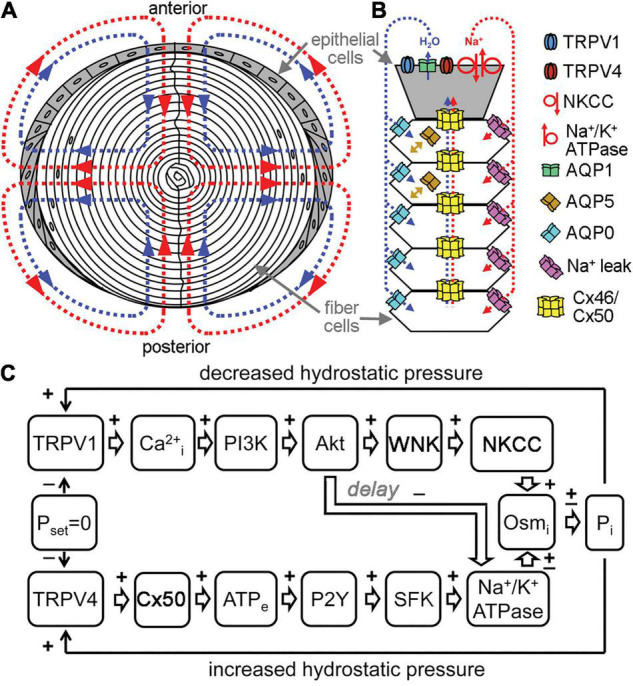
Channels regulate lens transport. **(A)** The flux of Na^+^ (red), followed by water (blue), enters the lens at both poles and exits at the equator and acts as a microcirculatory system. **(B)** Na^+^ flows into the lens though extracellular spaces, moves into fiber cells through Na^+^ leak channels, and flows back to the surface through gap junctions, where the Na^+^/K^+^-ATPase pumps it out of the lens. Water enters the lens through the extracellular spaces, moves into fiber cells through AQP0 and AQP5 driven by local osmotic gradients created by the transmembrane Na^+^ flux, and leaves the lens through AQP1 resulting from local osmotic gradients generated by the Na^+^/K^+^-ATPase. Hydrostatic pressure drives the water from cell to cell through gap junctions. **(C)** A feedback control mechanism maintains hydrostatic pressure (P) and water transport in the lens. Decreased pressure activates TRPV1, which then up regulates the NKCC and down regulates the Na^+^/K^+^-ATPase through a PI3K/Akt dependent pathway. Increases in pressure activate TRPV4, which then increases Na^+^/K^+^-ATPase activity through Cx50, ATP release and purinergic receptor activation of a Src family kinase (SFK).

The lens transport system has been described as an intricate microcirculation between cells (Mathias and Rae, 1985; Mathias et al., 2007). It is driven by a standing Na^+^ current and resulting water movement that direct flux inward at the poles and outward at the equator (Figure 2). Na^+^ and water enter the lens at the poles through the lens suture and move into the extracellular spaces between fiber cells. Influx is directed toward the lens sutures due to an extracellular diffusion barrier that has been identified in several species (Grey et al., 2003; Lim et al., 2009; Vaghefi et al., 2012). Although the molecular mechanisms of how this diffusion barrier is formed and regulated have yet to be elucidated, recent studies have begun to identify proteomic changes in the region of barrier formation (Wang et al., 2021). In the central lens, Na^+^ is taken up through leak channels that have not been fully characterized, but have been hypothesized to involve Cx46 hemichannels (Ebihara et al., 2014). Na^+^ then travels back to the lens equator through Cx46 and Cx50 gap junctions due to a difference in the resting potential between central and surface fiber cells. Finally, the Na^+^/K^+^-ATPase pumps Na^+^ ions out to the extracellular space against their gradient, and this also helps maintain ionic flux to the equator. Transport through the concomitant water gradient is facilitated by gap junctions and aquaporins (Gao et al., 2011). Changes in hydrostatic pressure and osmolarity can disrupt this network, leading to homeostatic responses that work to rebalance pressure and solute content. In a healthy lens, these main mediators are connexon channels, TRPV channels, and aquaporins (Gao et al., 2015; Delamere et al., 2020; Petrova et al., 2020).

The lens circulation is regulated by feedback control systems that modulate the activity of signaling enzymes, which in turn modify the activity of the channels and ionic transporters (Gao et al., 2015; Shahidullah et al., 2018, 2020; Delamere et al., 2020). The maintenance of steady Na^+^ flux results from the coordinated activity of the Na^+^/K^+^-ATPase, Na-K-2Cl co-transporter (NKCC), gap junctions and Na^+^ leak channels (Mathias et al., 2007; Shahidullah et al., 2020). The functional activity of the sodium transporters is maximal in the equatorial epithelial cells, while Na^+^ leak channel activity is highest in fiber cells (Gao et al., 2000; Candia and Zamudio, 2002; Tamiya et al., 2003; Mathias et al., 2007). Both the NKCC and Na^+^/K^+^-ATPase are implicated in responses to circulatory sodium homeostasis and hydrostatic pressure regulation, but through distinct mechanisms and responses (Delamere et al., 2020). Since the transmembrane movement of Na^+^ leads to concomitant water movement through osmosis, systems that regulate Na^+^ transport also lead to changes in water content in the lens.

Water movement helps maintain the lens’s optical properties by transporting nutrients and removing metabolic waste (Donaldson et al., 2001; Mathias et al., 2010). The main channels involved in water movement in the lens are aquaporins and gap junctions (Varadaraj et al., 2005; Gao et al., 2011). The distribution and type of aquaporin channels varies in each region of the lens, and these differences in aquaporin composition, and therefore water permeability, have been hypothesized to impact the magnitude and directionality of water flow (Schey et al., 2017). The principal aquaporins in the lens are AQP0, AQP1, and AQP5; with APQ1 localized to the epithelium, APQ 0 present in differentiating and mature fiber cells, and APQ5 found in all lens cell types (Stamer et al., 1994; Varadaraj et al., 2007; Petrova et al., 2015). When water enters the extracellular space, it moves into fiber cells through APQ0/5 and then is moved out of epithelial cells by APQ1/5, following the Na^+^ current. Since differences in hydrostatic pressure drive water through adjacent cells in the lens, aquaporin permeability likely plays a role in the magnitude of the hydrostatic pressure gradient. Knockout of APQ0 causes the formation of bilateral cataracts at an early age and a reduction in lens biomechanical load bearing at the lens sutures (Shiels et al., 2001; Kumari et al., 2015). APQ0 heterozygous KO also showed a reduction in load bearing and resulted in a cataract that arose later in development (Kumari et al., 2015). Knockout of APQ1 led to a three-fold reduction in water permeability at the lens epithelium; however, lens transparency and morphology was normal. When tested under conditions of metabolic and tensile stress, APQ1 KO mice developed a cataract (Ruiz-Ederra and Verkman, 2006). APQ5 KO, similarly to APQ1 KO, did not result in the presence of a cataract; however, metabolic and osmotic stress increased the chances of cataract formation (Kumari and Varadaraj, 2013). The movement of water through aquaporins is critical to the establishment of a pressure gradient in the lens, which drives the lens circulation.

Transient receptor potential vanilloid (TRPV) channels are also important osmoregulators in the lens that respond to changes in hydrostatic pressure by modulating sodium transport activity (Shahidullah et al., 2012, 2020; Delamere et al., 2020). TRPV1 and TRPV4 are the main lens TRP channels that detect negative and positive deviations in pressure, respectively, and reciprocally work to restore zero pressure (Shahidullah et al., 2012; Gao et al., 2015; Mandal et al., 2018). The TRPV1 response involves activation of PI3K/Akt signaling, which ultimately leads to an increase in NKCC activity, that increases the levels of intracellular sodium, therefore raising the intracellular pressure to combat the negative pressure stimulus. The TRPV1 dependent NKCC mechanism, however, is only active as a short-term response. If the negative pressure persists, then there is also a decrease of Na^+^/K^+^-ATPase activity (Sellitto et al., 2013; Delamere et al., 2020). In contrast, TRPV4 detects positive pressures in the lens and directs a P2Y/SFK dependent cascade that leads to an increase in Na^+^/K^+^-ATPase activity (Figure 2). This transports sodium out of epithelial cells to restore pressure (Shahidullah et al., 2012).

Gap junctions in the lens function in lens growth, development, and intercellular water transport (Mathias et al., 2010). The permeability and transport properties of these channels are implicated in lens epithelial growth and proliferation, Na^+^ and water coupled transport, Ca^2+^ transport, antioxidant gradients, and second messenger permeability (Mathias et al., 2010; Slavi et al., 2014; Brink et al., 2020). One important nutrient that connexin channels circulate is the tripeptide glutathione (GSH), which is the main alleviator of oxidative stress in lens fiber cells (Slavi et al., 2014; Braakhuis et al., 2019; Quan et al., 2021). The role of connexin channels in the lens has been primarily established using genetic knock-out (KO) and knock-in (KI) models (Berthoud et al., 2014). Measurement of the coupling conductance between lens fiber cells following genetic manipulation of connexin genes was used to elucidate the role of connexins in the microcirculation (Gong et al., 1998; Baldo et al., 2001; Martinez-Wittinghan et al., 2004). Knockouts of Cx50 and Cx46 had large impacts on lens function (Gong et al., 1997; White et al., 1998). However, KO of Cx43 failed to show significant differences in mutant and wild-type lenses (White et al., 2001; DeRosa et al., 2009). Cx50 knockout produced undersized lenses with a nuclear cataract (White et al., 1998; Rong et al., 2002). The Cx50 KO also had reduced coupling conductance in DF cells (Baldo et al., 2001). Cx46 KO showed reduced MF and DF coupling with the appearance of a dense central cataract, but normal lens size (Gong et al., 1998). Heterozygous Cx50 and Cx46 mutants showed reduced DF coupling for both, normal MF coupling for Cx50, and a 50% reduction in MF coupling for Cx46 (Gong et al., 1998; Baldo et al., 2001). Homozygous knock-in of Cx46 for Cx50 [Cx50(46/46)] produced small, but transparent, lenses with reduced DF coupling and increased MF coupling (White, 2002; Martinez-Wittinghan et al., 2003, 2004).

Analysis of Cx50(46/46) lenses using MRI imaging to visualize changes in anatomy and refractive index revealed that the knockin lenses had a reduced size, confirming a role for Cx50 in lens growth (Muir et al., 2020). Previous evidence showed Cx50 as a mediator in epithelial coupling and postnatal mitosis (Sellitto et al., 2004; White et al., 2007). Additionally, the Cx50(46/46) lenses had an increase in free water in the nucleus, reduction of the refractive index, and changes in lens geometry when compared to wild-type lenses (Muir et al., 2020). It had previously been shown that Cx50(46/46) lenses have an increased MF conductance, which established a reciprocal relationship between coupling conductance and hydrostatic pressure in the lens (Martinez-Wittinghan et al., 2004; Gao et al., 2011). These changes in hydrostatic pressure and coupling conductance indicate that gap junctions formed by Cx46 and Cx50 have differential roles in mediating water flow. The presence of Cx46 in fiber cells facilitates water flow, especially in the lens nucleus. The lens periphery and epithelial layer require Cx50 for normal growth and the directing of water and Na^+^ to the lens equator. The combined activity of both connexins is required for establishing both the directionality and magnitude of water and ion flux.

Each lens connexin has distinct properties in conductance, permeability, and function (White, 2003; Brink et al., 2020). Importantly for the microcirculation, Cx50 conductance is uniquely regulated by growth factor signaling (Shakespeare et al., 2009; Martinez et al., 2015). Growth factor signaling directs lens epithelial proliferation and differentiation, in part through the PI3K/Akt signaling pathway (Lovicu et al., 2011; Chaffee et al., 2016). When Akt was inhibited *in vitro*, a decrease in Cx50 conductance was observed (Martinez et al., 2015). PI3K/Akt signaling failed to modulate the activity of Cx46, suggesting specific targeting of Cx50 channels (Martinez et al., 2015). This selective regulation could differentially activate Cx50 conductance at the equator, where high levels of Cx50 conductance may be critical for directing the flux of Na^+^ to the equatorial epithelium.

Forces exerted on the lens through the zonules of Zinn have also been shown to modulate the microcirculation. Hydrostatic pressure changes in the lens have been reported to result from forces induced by the ciliary muscle and directly exerted on the lens by the zonules of Zinn (Chen et al., 2019). Changes in ciliary muscle contraction and relaxation conferred an increase or decrease in hydrostatic pressure, respectively, in the lens. Accommodative forces alter the shape and curvature of lenses, which changes the refractive index. Transient changes in hydrostatic pressure induced by input from the ciliary muscle could also cause changes to the refractive index in the absence of accommodation, by altering water content. The membrane localization of APQ5 was shown to be sensitive to changes in tension exerted by the ciliary muscle through the zonules (Petrova et al., 2020). Reducing zonular tension on the lens caused APQ5 to be relocated from the plasma membrane into the cytoplasm. The changes in localization are hypothesized to reduce water permeability and therefore alter the hydrostatic pressure in the equatorial zones where the zonules attach. Taken together, these studies suggest that the hydrostatic pressure gradient in the lens could regulated by the tension exerted by the ciliary muscle through the zonules, which could alter the absolute water content and refractive properties within the lens.

## Aging and Oxidative Stress Impact the Channels Underlying the Lens Circulation

Age and oxidative stress in the lens are usually coincident and lead to changes in the lens proteome, including connexin channel density and regulation (Gao et al., 2013; Gong et al., 2021; Quan et al., 2021). The lens is protected from oxidative damage by antioxidants, such as GSH, and chaperone proteins to prevent protein aggregation and dissolution from oxidative stress (Giblin, 2000; Lim et al., 2020). However, levels of available GSH decrease as a function of age, thereby increasing the amount of oxidized proteins and leading to disruptions in lens circulation and transparency (Ferrer et al., 1990; Wang et al., 2009). Oxidation of cysteine and methionine in crystallin proteins impact their solubility, aggregation, and refractive properties (Brennan et al., 2012). GPX-1 is involved in the glutathione redox cycle and GPX-1 knockout in mice lead to the formation of age dependent nuclear cataracts and a reduced number of functional Cx46 and Cx50 channels, implying that channel oxidation lowered the gap junctional coupling conductance of MF and DF cells (Reddy et al., 2001; Wang et al., 2009). Additional changes to lens proteins in mature fiber cells are due to age related posttranslational modifications (Lin et al., 1997; Ball et al., 2003; Korlimbinis et al., 2009). These modifications accumulate in many key protein mediators of the lens microcirculation, including connexins and aquaporins, impacting their activities (Korlimbinis et al., 2009; Wang and Schey, 2009; Slavi et al., 2016). Ultimately, aging and the concomitant reduction of antioxidants leads to failure of microcirculation and permanent impairment of vision.

Oxidation of lens proteins impacts lens transport and therefore transparency and refraction. Maintenance and delivery of antioxidants is crucial in ensuring proteins do not aggregate or accumulate defects from oxidation. This is especially relevant in the nucleus, where cells cannot produce their own antioxidant systems directly. GSH is the main lens antioxidant that prevents the accumulation of reactive oxygen species (Giblin, 2000; Lim et al., 2020). A GSH gradient exists, with the highest levels of GSH in the cortex and lowest in the nucleus. This gradient is due to the inability of MF cells in the nucleus to produce glutathione. MF cells rely on delivery of GSH through a concentration gradient that is propagated through water movement and involves Cx46 permeability (Slavi et al., 2014). Also, Cx46 was found to permeate GSH but not oxidized GSH (GSSG), making Cx46 a postulated channel in specific antioxidant delivery through diffusion to the nucleus. However, the mechanism of how GSSG is removed from the nucleus is still unknown, but is predicted to also work through passive diffusion to the surface cells or breakdown in the nucleus directly (Slavi et al., 2014). Connexin hemichannels opened in response to H_2_O_2_, mechanical stress, or ultraviolet radiation and allowed transit of glucose, GSH, and H_2_O_2_ (Liu et al., 2020; Quan et al., 2021). These results suggested that connexin hemichannels may also play a key role in maintaining the metabolic and antioxidative function of the lens, through facilitation of direct exchange of nutrients and redox metabolites between the extracellular space and cytoplasm. Additionally, aquaporins in the lens can transport hydrogen peroxide, and are hypothesized to maintain H_2_O_2_ levels that prevent oxidative damage but allow H_2_O_2_-mediated signaling (Varadaraj and Kumari, 2020). Levels of GSH and GPX-1 also decrease as a function of age, therefore increasing oxidative stress on lens fiber proteins (Ferrer et al., 1990; Spector et al., 2001; Lim et al., 2021).

Aging overall reduces the elasticity, refraction, and transparency of the lens, leading to loss of accommodation and vision (Cheng et al., 2019). The major age-related disorders of the lens are cataracts and presbyopia (Donaldson et al., 2017). Cataracts are opacities of the lens that cause a loss of refraction and pathologically impair sight (Shiels and Hejtmancik, 2016). Presbyopia is farsightedness caused by lack of accommodation from loss of lens elasticity (Van de Sompel et al., 2010). The lens’s impaired ability to maintain the microcirculation following protein oxidation leads to a depletion of nutrients and antioxidants, compounding the problems that produce failure of vision. Greater knowledge of the functions that these channels have in the microcirculation, refractive index, and overall lens homeostasis can help understand the mechanisms of presbyopia and cataract and potentially identify new therapies to delay their onset.

## Discussion

Connexins, TRPV channels, aquaporins, and sodium transporters are vital in maintaining lens homeostasis and optical properties. The lens microcirculation can be greatly influenced by the activity of any one of these channels, and maintaining the microcirculation can be difficult with the cumulative effects of aging. Connexon channels have functions in coupling cell conductance, ion and water transport, nutrient delivery, and lens growth. The recent discovery of TRPV channel activities in the lens gives more insight into how lens hydrostatic pressure is regulated through modulation of sodium transport activity. Aquaporins facilitate water movement in the lens and function in cell adhesion and shape, as well as nutrient maintenance and transport. Both APQ5 and TRPV channels respond to ciliary muscle input to restore hydrostatic pressure in the lens either by channel distribution or Na^+^ dependent responses, respectively. The specific mechanistic details of water content, transport, and regulation in the lens and its role in cataracts requires further study. MRI studies are a novel method for studying changes in lens water and dissolved protein content and provide a future direction for hydrostatic studies. Regulation of lens transport has expanded beyond its internal regulation systems, as novel discoveries show how it responds and adapts to different inputs from surrounding structures such as the ciliary body.

## Author Contributions

AG and TW wrote and edited the manuscript. LL and CS provided figures and edited the manuscript. All authors contributed to the article and approved the submitted version.

## Conflict of Interest

The authors declare that the research was conducted in the absence of any commercial or financial relationships that could be construed as a potential conflict of interest.

## Publisher’s Note

All claims expressed in this article are solely those of the authors and do not necessarily represent those of their affiliated organizations, or those of the publisher, the editors and the reviewers. Any product that may be evaluated in this article, or claim that may be made by its manufacturer, is not guaranteed or endorsed by the publisher.
